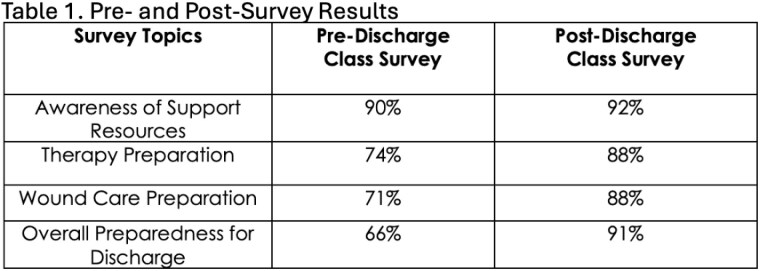# 804 Implementation of a Patient Discharge Class

**DOI:** 10.1093/jbcr/iraf019.335

**Published:** 2025-04-01

**Authors:** Lisa McMurtrey, Joanna Brooks, S Dyan Law, Alexander “Sasha” Trinkaus, Chelsea Gamero, Ronda Hopkins, Callie Thompson

**Affiliations:** University of Utah Health Burn Center; University of Utah Hospital; University of Utah Hospital; University of Utah Health Burn Center; University of Utah Health Burn Center; University of Utah Health Burn Center; University of Utah Health Burn Center

## Abstract

**Introduction:**

Our Burn Center admits approximately 350 patients per year with an average burn size of 18%. We serve a population that covers a large geographic area comprised of five states. Patients report that they face challenges as they prepare for discharge and transition to home. In discussion with burn survivors and their families, we identified that our patients/caregivers would benefit from attending a class to prepare them for the transition that will occur when they leave the hospital setting. We aimed to create a class centered on, and driven by, burn survivors’ needs.

**Methods:**

Our main patient education resource is a “Burn Care Guide for Patients and Families” (BCG). The BCG explains burn care treatment, roles of team members and psychosocial/reintegration support. It is available in electronic, printed, and Spanish formats. This resource served as a foundation for the content of the patient and caregiver discharge class. We co-produced this course with stakeholders by interviewing seven burn survivors/caregivers about how the transition process went for them and developing the course content based on their responses and expressed needs.

The class is taught by ten interdisciplinary team members which include nurses, social workers, and physical therapists. We utilized existing resources to develop and deploy the course. The class began in February 2024 and is taught weekly. Each member of our Burn Center team is empowered to discuss the class with their patients to encourage attendance. A pre-class survey is given to the patient/caregiver prior to the class. The post-class survey is given at the patient’s first clinic appointment following their discharge.

**Results:**

The class covers the following topics: things to expect, tips and tricks, insurance challenges, resources for support and concludes with time for questions. Sixty individuals have attended the class to date, twenty have completed a post-class survey. Of those, 90% felt it was an effective class. Table 1 shows the specific areas of improved effectiveness.

**Conclusions:**

We describe a co-produced patient discharge class that resulted in 91% of respondents feeling prepared to leave the hospital. This is a 25% increase from the level of preparedness reported prior to taking the course. This effective tool was successfully deployed with no additional resources.

**Applicability of Research to Practice:**

This class can serve as a template for other institutions to utilize with their patients/caregivers.

**Funding for the Study:**

N/A